# Experiencing authenticity – the core of student learning in clinical practice

**DOI:** 10.1007/s40037-016-0294-0

**Published:** 2016-09-15

**Authors:** Katri Manninen

**Affiliations:** Karolinska Institutet, Department of Learning, Informatics, Management and Ethics, Stockholm, Sweden

**Keywords:** Clinical practice, Learning, Pedagogical framework, Authenticity

## Abstract

**Introduction:**

Learning in clinical practice is challenging regarding organizational and pedagogical issues. Clinical education wards are one way to meet these challenges by focusing on both patient care and student learning. However, more knowledge is needed about how students’ learning can be enhanced and about patients’ and supervisors’ roles in these settings.

**Methods:**

The aim was to explore nursing students’ learning on a clinical education ward with an explicit pedagogical framework. Semi-structured interviews of students were analyzed using qualitative content analysis and an ethnographic study including observations and follow-up interviews of students, patients and supervisors was conducted.

**Results:**

The core of student meaningful learning experiences both external and internal authenticity. Students in early stages immediately created mutual relationships, experienced both external and internal authenticity, and patients became active participants in student learning. Without a mutual relationship, patients passively let students practice on their bodies. Students nearing graduation experienced only external authenticity, creating uncertainty as a threshold for learning. Caring for patients with complex needs helped students overcome the threshold and experience internal authenticity. Supervisors’ challenges were to balance patient care and student learning by working as a team. They supported students coping with the complex challenges on the ward.

**Discussion/Conclusion:**

Students need to experience external and internal authenticity to make learning meaningful. Experiencing authenticity, involving meaning-making processes and knowledge construction, is linked to transformative learning and overcoming thresholds. Therefore, an explicit pedagogical framework, based on patient-centredness, peer learning and the supervisory team, creates the prerequisites for experiencing external and internal authenticity.

## Introduction

In clinical settings the main focus is to provide patient care; education has a secondary role. This has been shown to jeopardize the possibilities for students to learn [1, 2]. Although the need for students to practise in clinical settings is acknowledged, this secondary role may result in insufficient organizational resources and the neglect of students’ educational support. Instead of helping students connect theoretical and practical knowledge and skills in patient-centred care, the risk is that supervisors focus on completing workload and controlling students [1]. Students also express loneliness and lack of support in their learning [2]. Most patients have a positive attitude regarding involvement in students’ learning, but sometimes they feel excluded from the communication between students and supervisors [3]. Clinical education wards have been established to enhance the integration of theory and practice, professional development and collaboration between clinical and educational settings. These wards train students at different levels and focus on inter-professional training or on one profession [4, 5].

The context for this thesis is a clinical education ward for nursing students at a university hospital in Sweden. The ward has eight beds and trains students at different levels. Fifteen students do their clinical practice simultaneously during a six-week placement. Five supervisors work on the ward, four are nurses and one is an assistant nurse. A physician and a senior clinical lecturer also serve as supervisors. A physiotherapist, dietician, occupational therapist and counsellor are linked to the ward. Patients are mainly admitted from the emergency department. The ward has a pedagogical framework based on an interpretation of Mezirow’s [6] theory of transformative learning. It consists of three parts: patient-centred learning, supervisors’ support and peer learning. Students are allowed to act as nurses; they work both individually and together with peers with support from the supervisors.

The focus of this research project was to explore and understand students’ learning in this specific context from multiple perspectives.

## Methods

Four studies were conducted, all with qualitative approaches based on constructivist and interpretative tradition [7], to explore nursing students’ learning in relation to encounters with patients, supervisors, peer students and other healthcare professionals. Data collection methods that were used were interviews and observations including field and reflective notes. The qualitative approaches allow an exploration of the phenomenon in its natural settings which means that the participants construct meanings of their experiences in interaction with other people, the researcher included. The researcher then makes an interpretation of the meanings resulting in co-constructed knowledge. Qualitative content analysis and ethnographic approach were used in the analysis.

In the first and second studies students’ experiences of their learning were explored using semi-structured individual and group interviews. The focus was on exploring how students experience their encounters with 1) patients, 2) supervisors, 3) peer students and 4) other healthcare professionals. Participants were asked to discuss and reflect on the encounters and the interviewer asked follow-up, probing and explanatory questions to obtain both broad and in-depth data. In the first study 19 first-year students were interviewed, and in the second study 18 final-year students were interviewed. Data from both studies were analyzed using qualitative content analysis focusing on the underlying meanings, i. e. the latent content of the data [8]. The findings from these two studies resulted in a need to explore interactions between students, patients and supervisors. Therefore in studies three and four an ethnographic approach [9] was used. Participant observations with follow-up interviews of 11 students, 10 patients and 5 supervisors, and a group interview of the supervisors were conducted. Study three focused on exploring patient-student encounters and in study four the focus was on exploring the pedagogical role of supervisors. During the observations extensive field notes including both observational and reflective notes were taken. Observational notes included description of activities and interactions, while reflective notes consisted of the observer’s thoughts and questions when observing. After each observation an individual follow-up interview was conducted. The participants were asked to talk about what had happened during the observation and how they felt about it. In the group interview of the supervisors they were asked to talk about their experiences and thoughts about supervision. Data from the observations were analyzed following an ethnographic approach which involves description, analysis and interpretation. This means describing and examining the relationships and linkages between the data from both observations and interviews and making an interpretation beyond the description [9].

## Results

The results show that the core of student meaningful learning is the experience of both external and internal authenticity. External authenticity refers to being in a real clinical setting meeting real patients. Internal authenticity is about the feeling of belonging and really contributing to patients’ health and well-being. The theory of transformative learning and the concepts of authenticity and threshold [6, 10, 11] were used to interpret the findings.

1. Students in the early stages of their education immediately created mutual relationships with patients and experienced both external and internal authenticity [12]. These mutual relationships became a basis for students’ learning. They also experienced belongingness which means a feeling of being a real part of the professional team taking care of patients. Students showed a patient-centred approach when learning.
2. For students nearing graduation, the learning process turned out to be more complex [13]. Students started out by expressing self-centredness and ambivalence in their relationships with patients, supervisors and peer students, which created uncertainty as a threshold for their learning. This resulted in experiencing only external authenticity. When the students took care of patients with extensive needs for nursing interventions they managed to overcome this threshold. They then experienced engagement consisting of creating mutual relationships and professional development, meaning that they also experienced internal authenticity.
3. Patients were always positively engaged in students learning but they were involved either actively or passively [14]. If the student created a good atmosphere and mutual relationships, the patient became an active participant in a joint action resulting in a learning relationship between them. If the student only created a one-way relationship, the patient became a passive participant resulting in an attending relationship. One-way relationships were characterized by students and patients communicating past each other. The patient became a training object; just lending a part of the body for the student to train on.
4. Supervisors’ approaches to and pedagogical role in students’ learning involved balancing patient care and student learning by being simultaneously patient- and student-centred [15]. This created a pedagogical challenge which they handled by working as a team and allowing students independence with support. Furthermore they made care plans for the patients and learning plans for the students following the clinical and pedagogical guidelines.

## Discussion

The findings show that authenticity has two dimensions, external and internal. Furthermore, experiencing authenticity is needed for meaningful and transformative learning. When students experience both external and internal authenticity, they make sense of their experiences and their learning becomes meaningful. These experiences involve challenges and need support to result in a process of meaning-making and knowledge construction. This process is also linked to transformative learning by Mezirow [6] and the findings of Land et al. [11] regarding learning in the liminal space, meaning that transformative learning involves overcoming thresholds. On a clinical education ward, characterized as the setting explored, transformative learning involves experiencing both external and internal authenticity, which forms the basis for creating learning relationships between patients and students. However, experiencing authenticity and mutual relationships is potentially troublesome and there are thresholds to overcome, particularly for students nearing graduation [6, 11]. Thus, supervisors need to acknowledge students’ different learning processes. The pedagogical framework is intended to help students overcome the thresholds, but accomplishing this depends on whether they understand and make use of the potential for learning. Students’ ability to think reflectively and to tolerate uncertainty is implied in the pedagogical framework. The supervisors’ role in the liminal space is crucial, as they provide both challenges and support. They identify students’ needs and help them through the transformative learning process by acknowledging the liminal space and troublesome knowledge [11]. Accordingly, supervisors need to have contextual knowledge, which is knowledge in different sciences tied to the context, which constitutes the pedagogical content knowledge needed to enhance student learning in clinical settings.

## Conclusion

The core of student learning in clinical practice is the experience of both internal and external authenticity. An explicit pedagogical framework based on patient-centredness, peer learning and the supervisory team creates prerequisites for experiencing authenticity.

## One piece of advice

To succeed with your thesis, have both short- and long-term goals with a step-by-step-plan to achieve them. Think SMART when you choose your goals as they should be specific, meaningful, accurate, realistic and time-bound. Discuss with your supervisors and peers and do not underestimate the power of reflection!
